# Hot Deformation Behavior, Dynamic Recrystallization and Phase Transformation Mechanism of Zr-Nb Alloy During Compression Processing

**DOI:** 10.3390/ma19153237

**Published:** 2026-07-30

**Authors:** Yuanbo Bi, Yuying Li

**Affiliations:** 1 Faculty of Mechanical and Electrical Engineering, Kunming University of Science and Technology, Kunming 650500, China; 2 School of Materials Science and Engineering, Tianjin University, Tianjin 300350, China; 3 School of Nuclear Science and Technology, University of South China, Hengyang City 421009, China; 15252283223@163.com

**Keywords:** hot compression, Zr-Nb alloy, flow stress, dynamic recrystallization, phase transformation

## Abstract

To reveal the high-temperature hot deformation mechanism and optimize the thermal processing window of Zr-Nb alloy, hot compression tests were implemented via a thermal simulation apparatus over temperatures ranging from 500 to 900 °C and strain rates of 0.01~10 s^−1^. The material’s flow performance, dynamic recrystallization (DRX) and phase transformation were systematically explored in this work. The results indicate that flow stress presents a negative correlation with deformation temperature and a positive correlation with strain rate. On the basis of measured stress–strain curves, the Arrhenius constitutive equation and processing map were established to quantitatively describe the alloy’s hot deformation behavior. Flow instability areas are mainly distributed in the low-temperature domain and the high strain rate region of medium-high temperature zones. The evolution of DRX under different strain rates and microstructural variations during phase transformation was analyzed in detail. Elevated strain rate leads to gradual grain refinement. Continuous dynamic recrystallization (CDRX) acts as the predominant DRX mode for Zr-Nb alloy, with merely minor discontinuous dynamic recrystallization (DDRX) features observed in the microstructure.

## 1. Introduction

Zirconium alloys are favored for nuclear fuel component fabrication in the nuclear energy industry owing to their superior corrosion resistance, low neutron absorption cross-section, and excellent high-temperature mechanical properties [1,2,3]. As core structural materials for nuclear fuel cladding tubes and reactor core boiling components, they can reliably isolate radioactive substances and maintain the stable and safe operation of nuclear reactors [4,5]. At room temperature, zirconium alloys exhibit a hexagonal close-packed (HCP) crystal structure. The insufficient number of operable slip systems significantly limits their plastic deformation capability at ambient conditions. Elevated deformation temperatures can activate multiple slip systems within the alloy matrix, which effectively ameliorates the material’s formability. The primary manufacturing processes for zirconium alloy components include hot extrusion, rolling, and solid-state welding between cladding tubes and end plugs [1,6]. Nevertheless, with the expanding application scope of zirconium alloys in advanced nuclear energy systems, the optimization of hot processing technologies to acquire superior high-strength service performance of zirconium alloys has become a critical research bottleneck that requires an urgent breakthrough.

Zirconium alloys exhibit highly sophisticated hot deformation responses under high-temperature service conditions. Hot compression testing enables the formulation of constitutive equations that describe the stress–strain correlation of materials under external loading, which facilitates the numerical prediction of deformation characteristics and spatial distributions of stress and strain during hot working procedures [7,8]. In recent years, emerging solid-state welding techniques represented by current-induced solid-state welding have been increasingly applied to the fabrication of nuclear fuel rod cladding tubes [1]. The developed constitutive models for zirconium alloys support precise characterization of material flow behaviors and grain refinement mechanisms across diverse weld zones. Furthermore, flow curves derived from hot compression experiments can be adopted to construct processing maps, which are capable of forecasting potential hot-working defects [9,10,11,12]. This theoretical and technical framework provides significant guidance for the performance enhancement of zirconium alloys and the optimization of industrial hot processing parameters.

Apart from macroscopic deformation and flow behavior, hot working of zirconium alloys is accompanied by dynamic recovery (DRV) and dynamic recrystallization (DRX). Once the temperature exceeds the phase transition threshold, phase transformation of the alloy can also be triggered. As a metallic material with high stacking fault energy (SFE), zirconium alloys preferentially undergo CDRX during thermal deformation. Nevertheless, CDRX is not the exclusive DRX mechanism under varied hot working parameters. Cui et al. [6] reported the coexistence of CDRX and DDRX in hot-compressed β-quenched Zr-4 alloy, and twin-associated DRX was also observed in their research. Accordingly, the dominant DRX mechanisms of zirconium alloys are highly dependent on hot processing conditions.

Specifically, once the hot working temperature surpasses the phase transformation initiation temperature $T_{\alpha \to \alpha +\beta }$, the alloy will generate β-phase with a body-centered cubic (BCC) structure. For zirconium alloys, the phase transformation initiation temperature $T_{\alpha \to \alpha +\beta }$ generally ranges from 550 to 850 °C, and the termination temperature $T_{\alpha +\beta \to \beta }$ falls within 700~950 °C. Such phase transition temperatures are strongly modulated by the composition ratio of alloying elements [13,14,15]. Notably, the operating temperature for solid-state welding of cladding tubes and end plugs is predominantly located in the α + β dual-phase region. Phase transformation occurring during hot processing is generally detrimental to the comprehensive service performance of nuclear cladding tubes. In this process, trace elements tend to redistribute and preferentially dissolve within the newly formed β-phase, disrupting the homogeneous distribution of secondary phases in the alloy matrix. These secondary phases are critical to the hydrogen absorption behavior, corrosion resistance and mechanical properties of zirconium alloys [16,17]. This adverse microstructural evolution is frequently overlooked when the α-phase remains the dominant constituent of the alloy microstructure.

Based on the above analysis, this paper innovatively establishes the constitutive equation and processing map of Zr-Nb alloy cladding tubes within the critical temperature range of solid-phase welding and systematically clarifies the dynamic recrystallization behavior and initial phase transformation mechanism of the material during thermal deformation. Different from conventional research on the thermal processing of zirconium alloys, this study focuses on the unique thermo-mechanical coupling effect of the novel zirconium alloy. It aims to improve and enhance the mechanical properties and thermal stability of the alloy under this innovative processing route, and the obtained processing windows can guide the adjustment of optimal welding parameters for pressure resistance welding of nuclear fuel rods. The research findings lay a solid theoretical foundation for the accurate simulation and prediction of the material deformation behavior under high-temperature and high-pressure service conditions. Furthermore, the results fill the research gap regarding the thermal deformation mechanism of zirconium alloys under current-assisted solid-phase welding, providing a significant scientific basis and technical support for the structural optimization, precision fabrication, and safe service of welding joints.

## 2. Materials and Methods

**Figure 1:**
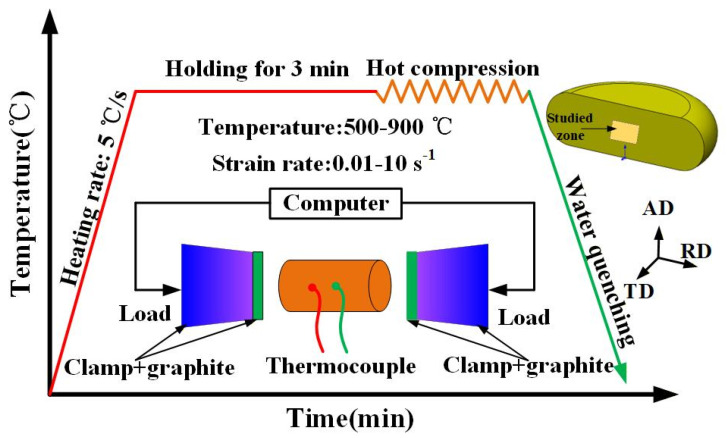
Schematic illustration of the hot compression process.

**Figure 2:**
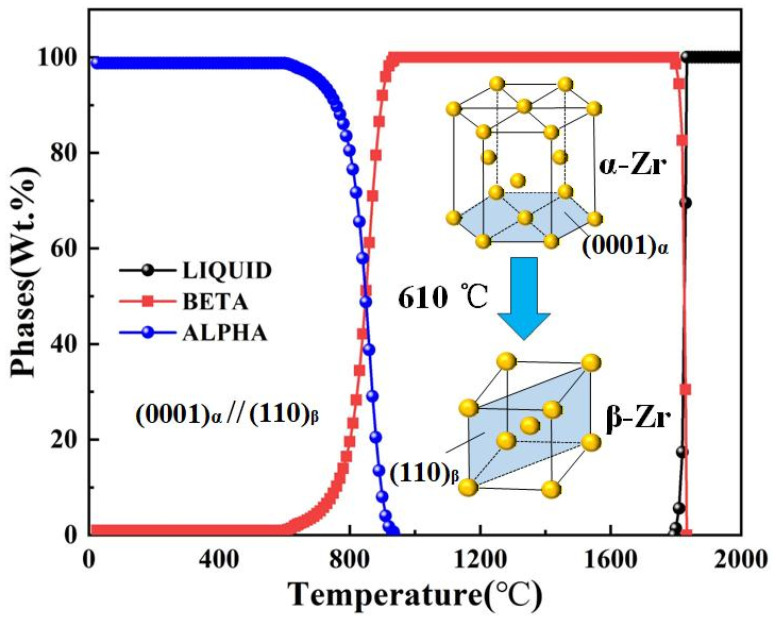
Phase diagram obtained by theoretical calculation.

The experimental material used in this work is a Zr-Nb alloy, and its chemical composition is listed in Table 1. Hot compression tests of the Zr-Nb alloy were conducted on a thermal simulation testing machine. To mitigate frictional contact between the specimen ends and the machine indenter during compression, graphite sheets were placed at all contact interfaces of the specimens and the indenter. Prior to compression deformation, the specimens were heated to target temperatures ranging from 500 to 900 °C at a constant heating rate of 5 °C/s, followed by a 3 min isothermal holding process to ensure uniform temperature distribution. The strain rate was set within the range of 0.01~10 s^−1^, and a fixed compression deformation amount of 70% was applied to all experimental specimens. Upon the completion of compression, immediate water quenching was performed on the deformed specimens to preserve the high-temperature deformed microstructure. The overall experimental procedure is illustrated in Figure 1. After hot compression, the cylindrical specimens were sectioned along the axial direction, and the central regions of the sectioned specimens were selected for microstructural characterization. The obtained samples were first mechanically ground and polished to achieve a scratch-free mirror surface, followed by argon ion polishing for further surface refinement. Additionally, the phase transformation diagram of the Zr-Nb alloy was calculated via the JMatPro software V14, as presented in Figure 2. The results indicate that the initial phase transformation temperature $T_{\alpha \to \alpha +\beta }$ of the tested Zr-Nb alloy is approximately 610 °C.

EBSD test specimens were further processed via argon ion polishing using an IM4000 ion milling system (Hitachi, Tokyo, Japan). The polishing process was performed at a working voltage of 4 kV and a polishing angle of 3° for a duration of 2 h. Microstructural observations were conducted utilizing a Zeiss Gemini 300 field emission scanning electron microscope (Carl Zeiss, Oberkochen, Germany) equipped with an Oxford C-nano EBSD detector (Oxford Instruments, Abingdon, UK) at an accelerating voltage of 20 kV. To further characterize the micromorphology and identify phase constituents accurately, transmission electron microscopy (JEM-F200, JEOL, Tokyo, Japan) testing was supplemented in this study. The Kearns factor is adopted to quantitatively characterize the texture features of the material. Its calculation formula is defined as $f=(f_{R},f_{T},f_{A})$, where $f_{R}$, $f_{T}$ and $f_{A}$ represent the intensity projection fractions of the basal plane along the rolling direction (RD), transverse direction (TD), and axial direction (AD), respectively, and the sum of the three values equals 1. Specifically, a random texture corresponds to a Kearns factor of $f_{R}=f_{T}=f_{A}$.

## 3. Results

### 3.1. Microstructure of Zr-Nb Alloy

**Figure 3:**
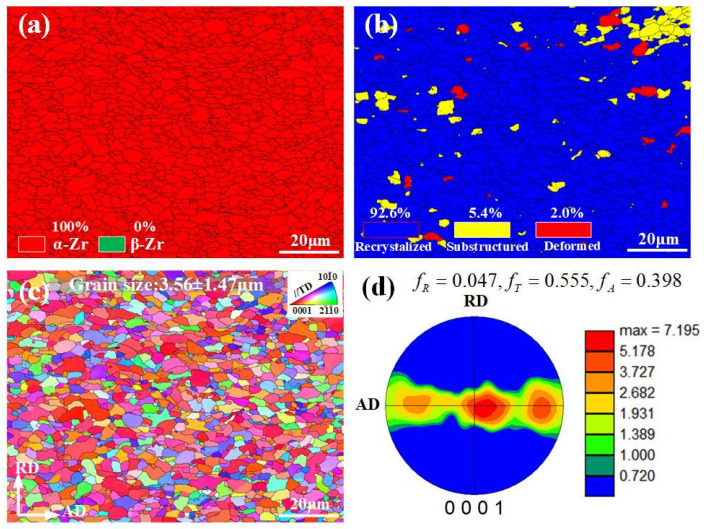
Initial microstructures of the Zr-Nb alloy prior to hot compression. (**a**) phase distribution map; (**b**) recrystallization map; (**c**) EBSD IPF orientation map; (**d**) pole figure.

**Figure 4:**
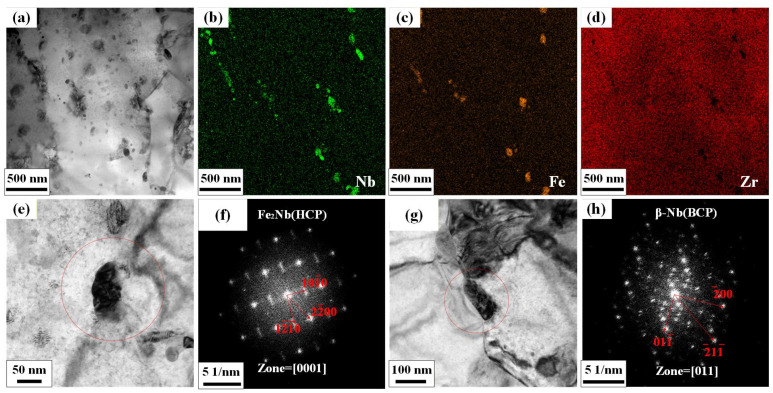
(**a**) TEM micrographs of Zr-Nb alloy, (**b**–**d**) element distribution, (**e**,**f**) TEM image and SAED pattern of Fe_2_Nb, (**g**,**h**) TEM image and SAED pattern of β-Nb.

Figure 3 presents the EBSD characterization results of the Zr-Nb alloy prior to hot compression. The phase distribution in Figure 3a reveals that the initial matrix microstructure consists entirely of the α phase. The recrystallization fraction map (Figure 3b) indicates that recrystallized grains account for 92.6% of the matrix structure. Figure 3c displays the EBSD inverse pole figure (IPF) map of the alloy matrix. The matrix grains exhibit an equiaxed morphology, with an average grain size of 3.56 ± 1.47 μm. The {0001} pole figure (PF) in Figure 3d features multiple intensity peaks along the axial direction (AD), demonstrating that most grains possess a <0001>//AD texture. Calculation of the Kearns factor derived from the {0001} pole figure further confirms that the material possesses a non-random crystallographic texture. Figure 4a shows the bright-field TEM image of the Zr-Nb alloy matrix, along with the corresponding selected area electron diffraction (SAED) patterns and elemental mapping results. Elemental distribution maps in Figure 4b–d demonstrate that the secondary phases primarily include Nb–Fe mixed phases and mononbium phases. To further identify the phase constituents, SAED indexing was performed. The phase was identified as hexagonal close-packed (HCP) Fe_2_Nb (Figure 4e,f), while the single niobium phase was verified as body-centered cubic (BCC) β-Nb (Figure 4g,h).

### 3.2. Material Flow Curve

**Figure 5:**
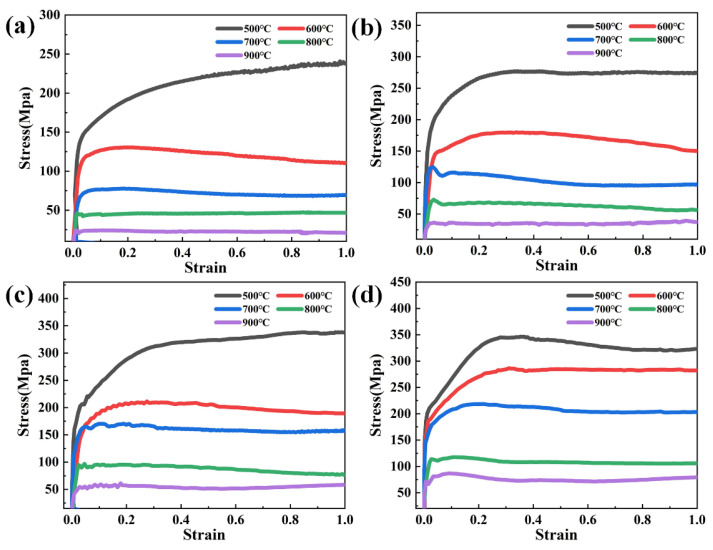
The stress–strain curves of the Zr-Nb alloy under different process parameters (**a**) $\dot{\varepsilon }$ = 0.01 s^−1^, (**b**) $\dot{\varepsilon }$ = 0.1 s^−1^, (**c**) $\dot{\varepsilon }$ = 1 s^−1^, and (**d**) $\dot{\varepsilon }$ = 10 s^−1^.

Figure 5 illustrates the compressive stress–strain responses of the Zr-Nb alloy at diverse temperatures and strain rates. Stress–strain profiles serve as a fundamental approach to analyzing the mechanical performance and plastic deformation behavior of metallic materials. As temperature rises, the softening effects such as DRV, DRX or phase transformation are enhanced, suppressing work hardening induced by dislocation pile-up and entanglement, and the flow stress drops evidently. With the progress of plastic deformation, geometrically necessary dislocations continuously accumulate and raise the total dislocation density inside the matrix. Higher dislocation density intensifies mutual dislocation obstruction and boosts the strain hardening rate; the extra resistance induced by dislocations further elevates instantaneous flow stress and dominates the evolution of flow stress during sustained deformation. Increasing the strain rate accelerates instantaneous plastic deformation, accompanied by a sharp rise in dislocation density and severe dislocation pile-up, which strengthens the work-hardening effect and ultimately yields higher flow stress. At the initial stage of deformation, rapid generation and accumulation of dislocations trigger a steep rise in flow stress with increasing strain, and work hardening dominates the overall plastic deformation behavior. Nevertheless, three distinct deformation characteristics emerge in the mid-to-late deformation stage under different thermomechanical conditions. Specifically, deformation at 500 °C with strain rates of 0.01 s^−1^ and 1 s^−1^ is predominantly controlled by work hardening, with negligible dynamic softening responses. For the deformation conditions of 500 °C/0.1 s^−1^ and 600 °C/10 s^−1^, the true stress–true strain curves exhibit a two-stage evolution, namely the work hardening stage and the steady-state equilibrium stage. By comparison, all other measured curves follow a three-stage evolutionary rule, including work hardening, dynamic softening, and final steady-state equilibrium. A noticeable upward warping phenomenon is observed in the flow curves when the strain exceeds 0.65. This abnormal stress increment is primarily attributed to intensified interfacial friction between the test specimen and equipment under large strain deformation, which induces a sharp rise in flow stress. Additionally, this phenomenon is closely associated with the dynamic evolution of microstructures during hot deformation.

Both the peak stress and steady-state stress exhibited a significant decreasing trend with the increase in deformation temperature: the average peak stress reached 314.9 MPa at 500 °C and decreased to 56.4 MPa at 900 °C, with a decrease rate of more than 82%, and the steady-state stress showed a consistent decreasing characteristic synchronously. Meanwhile, the difference between the peak stress and steady-state stress decreased continuously with the rise in temperature, which reflected the competitive mechanism that work hardening dominated at low temperatures and the dynamic softening effect was enhanced at high temperatures. The peak stress had a higher sensitivity to temperature changes than the steady-state stress, which demonstrated the strong temperature dependence of work hardening in the initial deformation stage and the stress stability after the dynamic balance between work hardening and dynamic softening was achieved in the steady-state stage.

### 3.3. Constitutive Equation and Activation Energy

Deformation temperature, strain rate and strain exert significant influences on the flow stress of deformed materials. The empirical formula based on the Arrhenius model [18] is well applied to describe the relationship among flow stress, deformation temperature and strain rate. The governing equation is given below:

(1) $$\dot{\varepsilon }=A_{1}\sigma^{n_{1}}\exp(-\frac{Q}{RT})\left(\alpha \sigma <0.8\right)$$

(2) $$\dot{\varepsilon }=A_{2}[\exp(\beta \sigma )]\exp(-\frac{Q}{RT})\left(\alpha \sigma >0.8\right)$$

(3) $$\dot{\varepsilon }=A{\left[\sinh(\alpha \sigma )\right]}^{n}\exp(-\frac{Q}{RT})$$

where $\dot{\varepsilon }$ denotes the strain rate, σ represents the flow stress (MPa), and R is the universal gas constant with a value of 8.314 J/(mol·K). A1, A2, A, α and β stand for material constants, Q is the deformation activation energy (kJ/mol), n_1_ and n refer to stress exponents, and T is the absolute temperature.

The coefficient α can be determined via the equation below:

(4) $$\alpha =\frac{\beta }{n_{1}}$$

The natural logarithm of Equation (1) is taken to obtain the subsequent formula:

(5) $$\ln\dot{\varepsilon }=\ln A_{1}+n_{1}\ln\sigma -\frac{Q}{RT}$$

The natural logarithm of Equation (2) is taken to obtain the subsequent formula:

(6) $$\ln\dot{\varepsilon }=\ln A_{2}+\beta \sigma -\frac{Q}{RT}$$

The natural logarithm of Equation (3) is taken to obtain the subsequent formula:

(7) $$\dot{\varepsilon }=\ln A_{3}+n\ln[\sinh(\alpha \sigma )]-\frac{Q}{RT}$$

**Figure 6:**
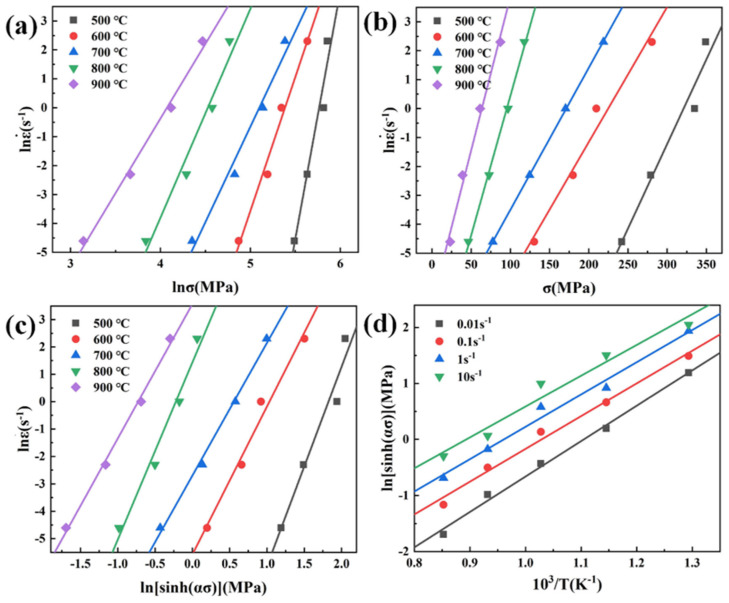
The linearly fitted relationship between: (**a**) $\ln\dot{\varepsilon }-\ln\sigma$, (**b**) $\ln\dot{\varepsilon }-\sigma$, (**c**) $\ln\dot{\varepsilon }-\ln[\sinh(\alpha \sigma )]$, (**d**) $\ln[\sinh(\alpha \sigma )]-10^{3}/T$.

To explore the correlation between flow stress and thermal deformation parameters, the peak stress $\sigma_{p}$ corresponding to each deformation condition is selected as the research object for subsequent analysis. Linear fitting is performed on $\ln\dot{\varepsilon }-\ln\sigma$ and $\ln\dot{\varepsilon }-\sigma$ respectively, and the fitting results are presented in Figure 6a,b. Based on the average slope values of the fitted straight lines, the corresponding values of $n_{1}$ and β are determined as $n_{1}$ = 9.066818, β = 0.071364, $\alpha =\frac{\beta }{n_{1}}=0.00787091$. Subsequently, the deformation activation energy Q can be calculated via Equation (7).

(8) $$Q=R\frac{\partial \ln\dot{\varepsilon }}{\partial \ln[\sinh(\alpha \sigma )]}\frac{\partial \ln(\sinh(\alpha \sigma ))}{\partial (1000/T)}$$

Corresponding linear fitting analyses are implemented for the $\ln\dot{\varepsilon }-\ln[\sinh(\alpha \sigma )]$ and $\ln[\sinh(\alpha \sigma )]-10^{3}/T$, with the fitting outcomes displayed in Figure 6c,d. Substituting the linear fitting results into Equation (8) yields the values of the n and Q as 5.779984 and 281.5228 kJ/mol, respectively.

Zener and Hollomon proposed the Z parameter to characterize the combined effects of strain rate and deformation temperature on material deformation, and its mathematical expression is defined as follows:

(9) $$Z=\dot{\varepsilon }\exp(\frac{Q}{RT})=A{\left[\sinh(\alpha \sigma )\right]}^{n}$$

The logarithm of (9) is:

(10) lnZ=lnA+nln[sinh(ασ)]

Additionally, the Z parameter serves as a valid indicator for verifying the reliability of the established constitutive equation. The aforementioned formula can be rearranged into the alternative form below:

(11) $$\sigma =\frac{1}{\alpha }\ln\{{\left(\frac{Z}{A}\right)}^{\frac{1}{n}}+{\left[{\left(\frac{Z}{A}\right)}^{\frac{2}{n}}+1\right]}^{\frac{1}{2}}\}$$

**Figure 7:**
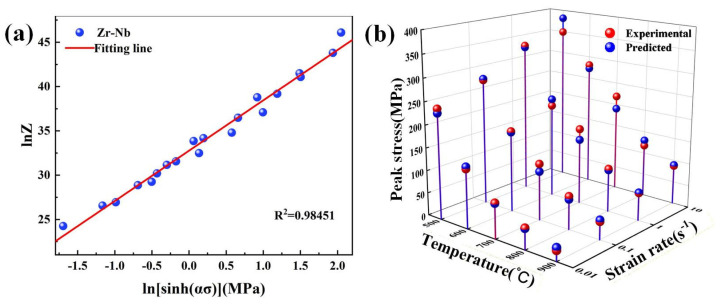
(**a**) The linearly fitted relationship of lnZ−ln[sinh(ασ)], (**b**) experimental and predicted peak stress values.

Using the Q value calculated from the above formula, the Z values under varying temperatures T and strain rates $\dot{\varepsilon }$ are acquired through parameter substitution. On this basis, the functional relationship between ln Z and ln[sinh(ασ)] is plotted. As illustrated in Figure 7a, the intercept of the fitted straight line corresponds to ln A, yielding a parameter A of 1.69693 × 10^14^. A high correlation coefficient of R = 0.98451 is observed from the fitting results in Figure 7a. Furthermore, the predicted and experimental flow stress values derived from Equation (11) are compared in Figure 7b. The excellent consistency between predicted and experimental data demonstrates the high reliability of the established constitutive model for the tested material. By substituting all the calculated parameters into Equation (3), the final constitutive equation is expressed as follows:

(12) $$\dot{\varepsilon }=1.69693\times 10^{14}{\left[\sinh(0.00787091\sigma )\right]}^{5.779984}\exp(\frac{-281522.8}{RT})$$

Equation (12) can also be transformed into the following equation:

(13) $$\sigma =\frac{1}{0.00787091}\ln\{{\left(\frac{Z}{1.69693\times 10^{14}}\right)}^{\frac{1}{5.779984}}+{\left[{\left(\frac{Z}{1.69693\times 10^{14}}\right)}^{\frac{2}{5.779984}}+1\right]}^{\frac{1}{2}}\}$$

### 3.4. Processing Map

Processing maps are capable of forecasting material instability during hot working and identifying potential unstable regions throughout thermal processing. In the hot deformation process, the power dissipation coefficient (η) is defined as the ratio of the dissipative covariance J to the linear dissipation $J_{\max}$:

(14) $$\eta =\frac{J}{J_{\max}}=\frac{2m}{m+1}$$

Linear dissipation $J_{\max}$ can be mathematically formulated as:

(15) $$J_{\max}=\frac{\sigma \dot{\varepsilon }}{2}$$

The expression of the dissipation covariance J is as follows:

(16) $$J=\frac{m}{m+1}\sigma \dot{\varepsilon }$$

The strain rate sensitivity index m can be expressed as:

(17) $$m=\frac{\dot{\varepsilon }\partial \sigma }{\sigma \partial \dot{\varepsilon }}=\frac{\partial (\ln\sigma )}{\partial (\ln\dot{\varepsilon })}$$

To precisely predict the occurrence of flow instability, the rheological instability criterion for the investigated material is defined as follows:

(18) $$\xi (\dot{\varepsilon })=\frac{\partial \ln(\frac{m}{m+1})}{\partial \ln\dot{\varepsilon }}+m<0$$

$\xi (\dot{\varepsilon })$ represents the instability parameter, when $\xi (\dot{\varepsilon })$ < 0, to precisely predict the occurrence of flow instability, the rheological instability criterion for the investigated material is defined as follows. A satisfied instability condition indicates that the corresponding processing parameters lie in an unstable regime, which renders the material susceptible to hot-working defects.

**Figure 8:**
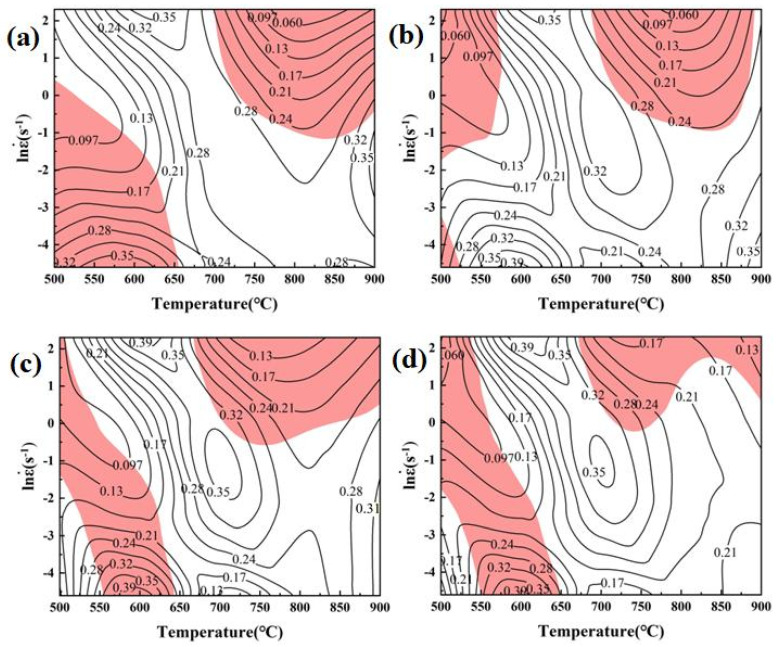
Processing maps of the Zr-Nb alloy obtained at true strains: (**a**) 0.3, (**b**) 0.45, (**c**) 0.6, (**d**) 0.75.

Figure 8a–d present the processing maps of the Zr-Nb alloy at true strains of 0.3, 0.45, 0.6, and 0.75, respectively. The contour values in the maps denote the power dissipation factor η, while the dark red shaded areas represent the instability domains where metallurgical defects such as voids and cracks tend to initiate and propagate. At a true strain of 0.3, the instability domains cover two parameter regions: 500~650 °C/0.01~1 s^−1^ and 725~900 °C/1~10 s^−1^. When the true strain is 0.45, the instability zone is 500~525 °C/0.01~0.1 s^−1^, 500~575 °C/0.1~10 s^−1^, 725~875 °C/1~10 s^−1^. When the true strain is 0.6, the instability zone is 500~550 °C/0.1~10 s^−1^, 550~650 °C/0.01~0.1 s^−1^, 700~900 °C/1~10 s^−1^. When the true strain is 0.75, the instability zone is 500~550 °C/0.1~10 s^−1^, 550~650 °C/0.01~0.1 s^−1^, 700~900 °C/1~10 s^−1^. All regions outside the aforementioned instability domains are regarded as safe processing windows. Overall, the unstable domains are predominantly distributed in low-temperature zones and high-strain-rate zones within the medium-to-high temperature range. Accordingly, the optimal hot processing window for the Zr-Nb alloy is determined to be 650~900 °C with a strain rate of 0.01~1 s^−1^.

## 4. Discussion

### 4.1. Microstructural Evolution with Strain Rate

**Figure 9:**
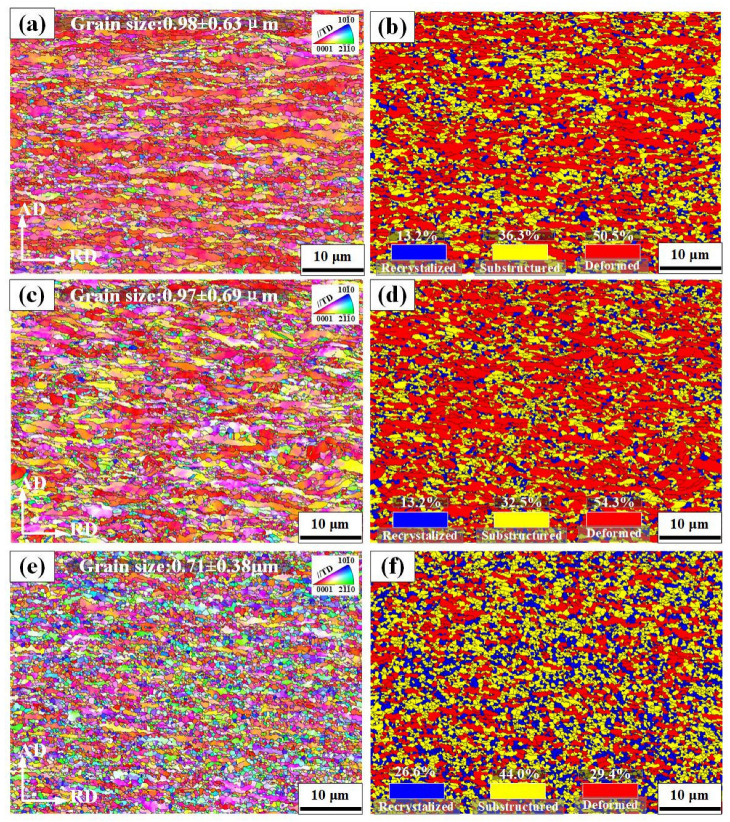
(**a**,**c**,**e**) IPF maps and (**b**,**d**,**f**) recrystallization maps of Zr-Nb alloy after hot compression at a temperature of 500 °C: (**a**,**b**) 0.1 s^−1^, (**c**,**d**) 1 s^−1^, (**e**,**f**) 10 s^−1^.

Figure 9 displays the inverse pole figure (IPF) maps characterizing the microstructural morphology of the compressed Zr-Nb alloy at 500 °C under different strain rates. Microstructural observation reveals that all specimens consist of elongated grains aligned along the rolling direction (RD) alongside numerous equiaxed grains. As illustrated in Figure 9a,c, the average grain sizes are 0.98 ± 0.63 μm and 0.97 ± 0.69 μm at the strain rates of 0.1 s^−1^ and 1 s^−1^, respectively, showing a negligible difference in grain dimension. By contrast, the specimen deformed at a higher strain rate of 10 s^−1^ exhibits an average grain size of 0.97 ± 0.69 μm (Figure 9e), with prominent grain refinement compared with specimens under low strain rate conditions. The underlying mechanism is that high strain rates suppress grain boundary migration and subgrain growth, ultimately contributing to the formation of refined microstructures. At the strain rates of 0.1 s^−1^ and 1 s^−1^, the dynamic recrystallization (DRX) volume fraction is measured to be 13.2%, as presented in Figure 9b,d. Specimens deformed at 0.1 s^−1^ possess abundant substructures, demonstrating that low strain rates facilitate dynamic recovery (DRV). In comparison, the DRX volume fraction peaks at 26.6% under the strain rate of 10 s^−1^ (Figure 9f). In general, high strain rates elevate the dislocation density induced by thermal deformation while shortening the duration for HABs migration and dislocation motion, thereby inhibiting both DRV and DRX behaviors. Nevertheless, DRX during hot compression is a non-instantaneous process. Early nucleation and growth of recrystallized grains can continuously accommodate ongoing deformation, rendering the actual DRX fraction higher than the statistically measured value. This microstructural phenomenon has been reported in previous studies [7]. Accordingly, the dependence of DRX fraction on strain rate varies across different metallic materials and compressed specimens with distinct specifications.

**Figure 10:**
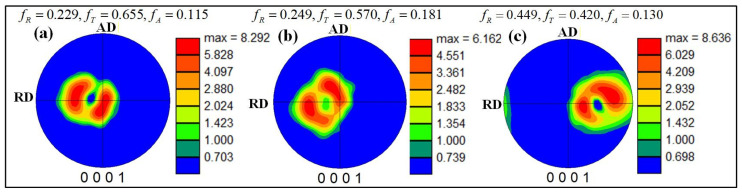
Pole figures of the Zr-Nb alloy after hot compression at a temperature of 500 °C: (**a**) 0.1 s^−1^, (**b**) 1 s^−1^, (**c**) 10 s^−1^.

Figure 10 shows the {0001} pole figure (PF) of Zr-Nb alloy. It can be seen from Figure 10a,b that when the strain rate is 0.1 s^−1^ and 1 s^−1^, $f_{T}$ are 0.655 and 0.570, respectively. Most of the grains show the texture characteristics of <0001>//TD; the texture intensity reaches the maximum value at 0.1 s^−1^. With the increase in strain rate, $f_{T}$ gradually decreases and $f_{R}$ gradually increases. When the strain rate is 10 s^−1^, it is 0.449 (Figure 10c), and the proportion of grains <0001>//RD is the largest. Through analysis, it can be found that the texture characteristics first change from <0001>//AD of the base metal to <0001>//TD, and with the increase in strain rate, it changes to <0001>//RD.

**Figure 11:**
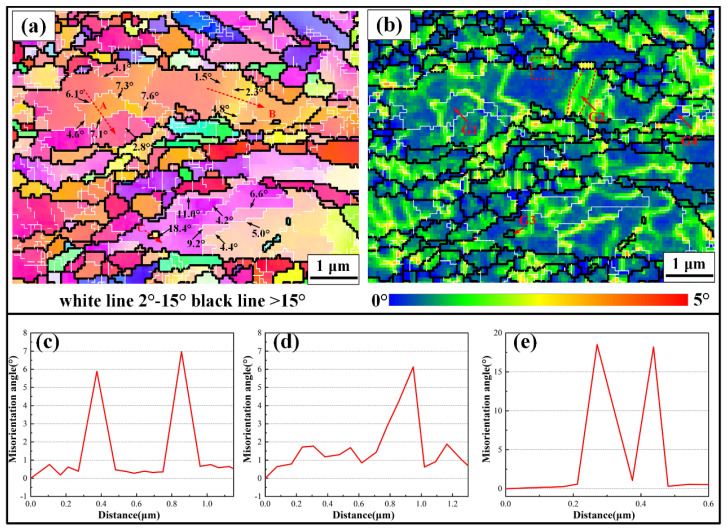
A partial enlarged view of Figure 8c: (**a**) IPF maps, (**b**) KAM distribution maps, (**c**) line A, (**d**) line B, (**e**) line C.

Figure 11 shows the enlarged EBSD micrographs of the Zr-Nb alloy deformed at 500 °C with a strain rate of 1 s^−1^. As observed in Figure 11a, Grain G1 is entirely enclosed by LAGBs. Scan Line A traverses Grain G1, and two misorientation peaks emerge at the boundaries of G1 (Figure 11c), with corresponding misorientation angles of 6.1° and 7.1°, respectively. These LAGBs originate from the DRV process and have the potential to transform into HAGBs via CDRX [19,20]. Distinct color contrast is identified between Grain G2 and its surrounding matrix, accompanied by intermittent grain boundaries with misorientation angles ranging from 1.5° to 2.3°. For Scan Line B crossing Grain G2, misorientation peaks are detected at discontinuous LAGBs away from the interior of G2. Combined with the kernel average misorientation (KAM) map in Figure 11b, the red-lined boundaries within Grain G2 exhibit high dislocation density. Such boundaries tend to absorb adjacent dislocations through DRV, ultimately evolving into continuous LAGBs. Grain G3 is a fine grain embedded within a coarse parent grain, and its entire grain boundary consists of HAGBs. Scan Line C across Grain G3 also captures two misorientation peaks at the grain boundary, with an orientation difference of 18°, which deviates substantially from the misorientation range of LAGBs (2–15°). This structural feature indicates that Grain G3 is developed through the CDRX mechanism. In contrast, Grain G4 is bounded by a mixture of LAGBs and discontinuous HAGBs, demonstrating that its CDRX microstructural evolution remains incomplete. Additionally, prominent grain boundary bowing is observed in several grains. Generally, grain boundary bowing is recognized as a precursor to DDRX nucleation, though this morphological feature alone cannot fully confirm the occurrence of DDRX. Typical DDRX characteristics involve a distinct dislocation density gradient across bowed boundaries, with grain boundaries migrating from low-dislocation-density regions toward high-dislocation-density areas [21,22]. Most bowed grain boundaries in Figure 11 fail to satisfy this criterion. Only a limited number of grains (marked by the red frame in Figure 11b) exhibit features consistent with the DDRX mechanism, verifying that CDRX acts as the dominant DRX mode for the investigated Zr-Nb alloy.

**Figure 12:**
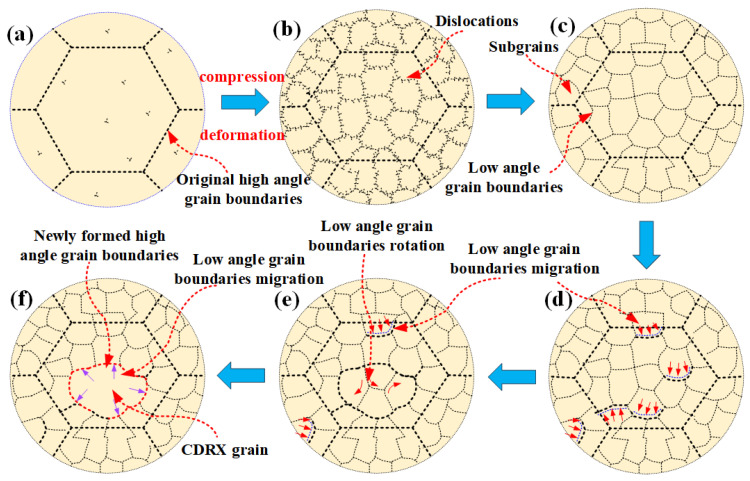
The forming process of CDRX.

The Zr-Nb alloy possesses a low initial dislocation density, as displayed in Figure 12a. Intense plastic deformation induced by hot compression substantially proliferates internal dislocations within the material. At elevated temperatures, dislocations readily undergo slip and climb motions. Dislocations distributed on identical slip planes offset one another and undergo polygonization, ultimately generating dense dislocation cell structures (Figure 12b). Continuous migration of deformation-induced dislocations enables adjacent subgrain boundaries with minor misorientation angles to coalesce into high-misorientation subgrain boundaries, constructing interconnected subgrain boundary networks inside grains (Figure 12c). These subgrain boundaries constantly capture newly generated intragranular dislocations to elevate their misorientation magnitude, triggering subgrain boundary migration during ongoing deformation (Figure 12d), followed by subgrain rotation behavior (Figure 12e). Ultimately, subgrain boundaries transform into HAGBs once the subgrain misorientation accumulates to 15°, completing the CDRX microstructural evolution process (Figure 12f) [6,12,13].

### 4.2. Microstructural Evolution with Deformation Temperature

**Figure 13:**
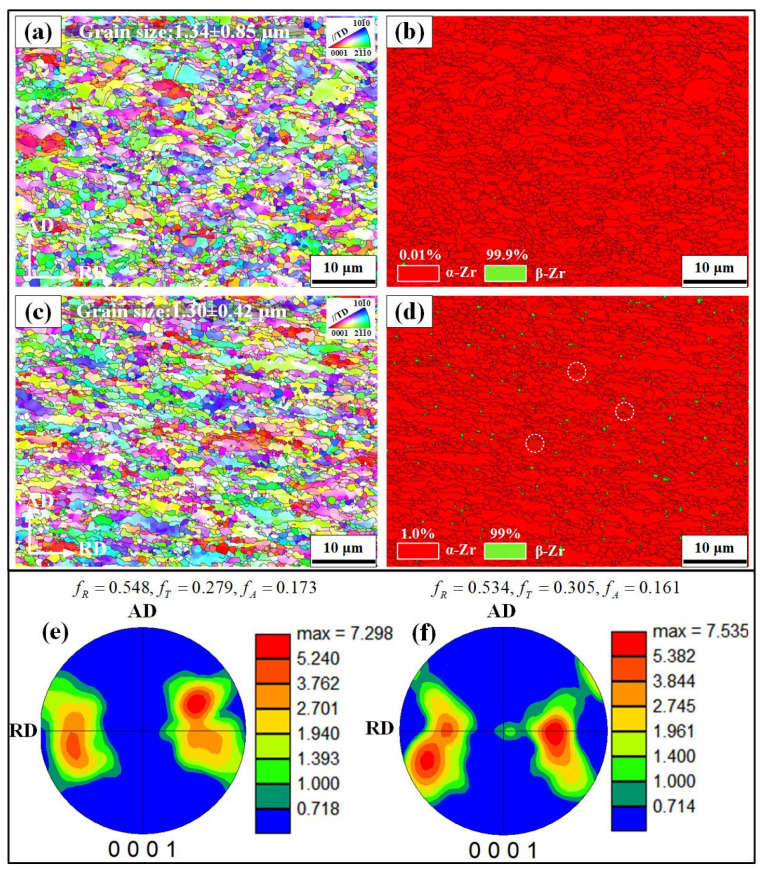
(**a**,**c**) IPF maps, (**b**,**d**) phases map and (**e**,**f**) pole figure of Zr-Nb alloy after hot compression at the strain rate of 10 s^−1^: (**a**,**c,e**) 600 °C, (**b**,**d,f**) 700 °C.

Figure 13a,c present the EBSD maps of the Zr-Nb alloy deformed at a constant strain rate of 10 s^−1^ under temperatures of 600 °C and 700 °C, respectively. At 600 °C, the microstructure contains abundant grains aligned parallel to the rolling direction (RD), consisting of numerous fine grains and a certain proportion of roughly equiaxed coarse grains. Elevating the deformation temperature promotes the progressive growth of recrystallized grains. Compared with the microstructure obtained at 500 °C (Figure 9e), the average grain size increases to 1.34 ± 0.85 μm at 600 °C (Figure 13a). Given that this deformation temperature is close to the theoretical phase transformation initiation temperature of 610 °C (Figure 2), only sporadic β-grain regions are detected in Figure 13b. Accordingly, grain refinement under this condition is predominantly governed by the dynamic recrystallization of α grains. At 700 °C, the alloy fully resides within the α + β two-phase field. With the growth of recrystallized α grains and the enhanced DRX extent, abundant fine α grains are generated, while phase transformation simultaneously produces a certain number of fine β grains. The corresponding average grain size is measured as 1.30 ± 0.42 μm (Figure 13c). As illustrated in Figure 13d, the majority of β grains distribute along grain boundaries, with only a small fraction located within the grain interior, as marked by the white dashed circles. Furthermore, according to the data in Figure 13e,f, the texture $f_{R}$ indices at 600 °C and 700 °C are 0.548 and 0.534, respectively, demonstrating that the microstructures under both temperatures are dominated by a <0001>//RD preferred orientation.

**Figure 14:**
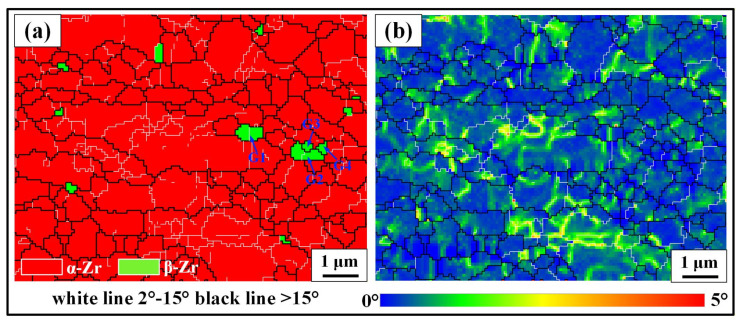
A partial enlarged view of Figure 13d: (**a**) Phase map, (**b**) KAM distribution map.

Figure 14 presents the magnified EBSD micrograph of the alloy specimen deformed at a strain rate of 10 s^−1^ and a temperature of 700 °C. As illustrated in Figure 14a, LAGBs are predominantly distributed within coarse grains, demonstrating the activation of DRV and DRX within these coarse grain domains. The majority of secondary β grains nucleate and distribute along grain boundaries. The high-density lattice defects accumulated at grain boundaries effectively lower the critical nucleation energy, thereby facilitating the preferential precipitation of grain-boundary β phases. Within the α + β two-phase field, primary β grains undergo synchronous deformation with α grains during hot compression. Noticeable LAGBs are detected inside the primary β grains (exemplified by Grain G1), which originate from dislocation migration driven by hot compressive deformation. Owing to the abundant slip systems in the body-centered cubic (BCC) structured β phase, dislocations within the β lattice possess enhanced mobility in the forms of slip and climb during plastic deformation. As displayed in Figure 14b, the maximum dislocation density is concentrated in the vicinity of LAGBs. Hot deformation is a competitive process involving continuous dislocation multiplication and accumulation, as well as dislocation annihilation induced by DRV, DRX, and grain growth. This competing mechanism results in substantially reduced local misorientation in numerous α grains. Grains G2 and G3 are fully recrystallized β grains bounded by HAGBs, which provides solid evidence for the rapid growth and aggregation of newly nucleated β grains or the occurrence of complete DRX.

### 4.3. Tem Analysis Before and After Theoretical Phase Transformation Temperature

**Figure 15:**
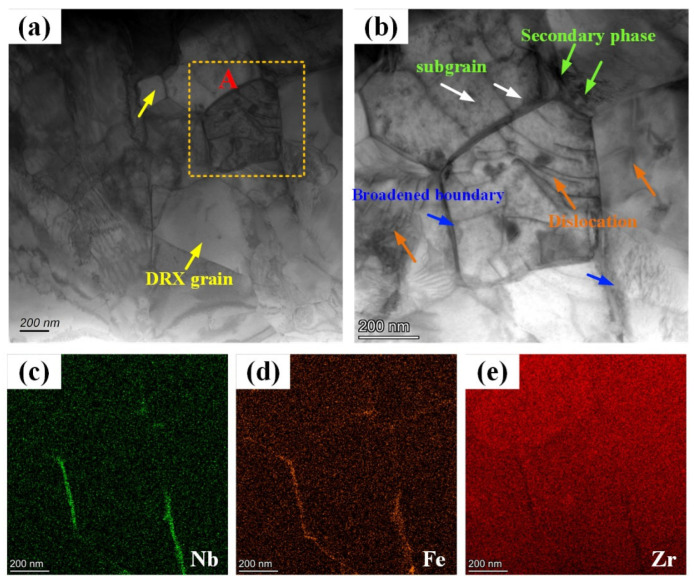
TEM micrographs of Zr-Nb alloy at a temperature of 600 °C and a strain rate of 10 s^−1^: (**a**,**b**) bright-field microscopic images, (**c**–**e**) the surface scanning results of Figure 15b.

Figure 15 displays TEM bright-field micrographs of the alloy subjected to hot deformation at 600 °C under a strain rate of 10 s^−1^. It is evident from Figure 15a that a high density of dislocations is prevalent inside most grains, with only a small fraction of recrystallized grains identified, as highlighted by the yellow arrows. Figure 15b provides a magnified view of Region A in Figure 15a, where distinct subgrain structures are observed in partial grains, and dislocation clusters are concentrated along subgrain boundaries. Additionally, several grains exhibit widened grain boundaries. Combined with the elemental distribution results presented in Figure 15c–e, the widened grain boundaries can be categorized into two distinct types. The first type corresponds to grain boundaries enriched with Nb and Fe elements, while the second type features no obvious elemental segregation. The broadening of element-free grain boundaries is primarily attributed to the intensive accumulation of dislocations at boundary sites, as reported in previous studies [6]. Grain boundaries serve as effective dislocation sinks owing to their increased free volume, which promotes grain boundary bowing during thermal deformation [23]. Elemental mapping results in Figure 15c–e confirm pronounced diffusion-induced segregation of alloying elements at triple-grain junctions and adjacent extended grain boundary zones. As typical β-phase stabilizers, Nb and Fe are capable of broadening the temperature range of the β-phase field [24,25]. The diffusion kinetics of these two alloying elements further modulate the phase transformation behavior, as well as the nucleation and growth kinetics of newly formed grains. Moreover, elemental characterization verifies that the majority of secondary phases within the grain interior have fully dissolved after hot deformation, while only a limited number of residual secondary phases remain and are distributed exclusively in the near-boundary regions, as consistent with the morphological features in Figure 15b.

**Figure 16:**
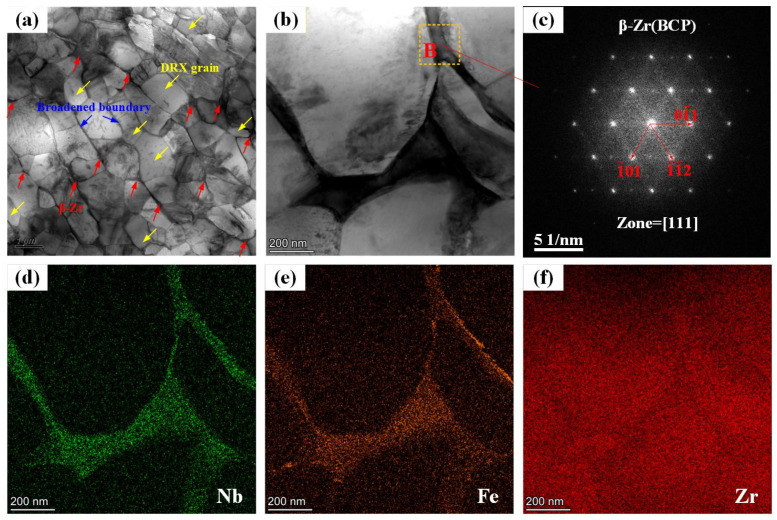
TEM micrographs of Zr-Nb alloy at a temperature of 700 °C and a strain rate of 10 s^−1^: (**a**) and (**b**) bright-field microscopic images, (**c**) SAED pattern, (**d**–**f**) the surface scanning results of Figure 16b.

Figure 16 illustrates bright-field TEM micrographs of the alloy deformed at 700 °C with a strain rate of 10 s^−1^. A large number of DRX grains can be clearly identified in Figure 16a, as marked by yellow arrows. Elevated deformation temperatures diminish the stored distortion energy within the strained alloy, yet significantly enhance the grain boundary sliding capacity, which acts as a dominant factor in microstructural evolution. This temperature-dependent effect accelerates the nucleation and growth kinetics of new grains, demonstrating that high-temperature conditions facilitate the progression of DRX, which is consistent with previous research findings [26,27]. Combined with the elemental distribution data in Figure 16d–f, sufficient diffusion of alloying elements occurs at high temperatures, leading to the complete dissolution of secondary phases previously precipitated within α grains. In comparison with the microstructure deformed at 600 °C, more prominent segregation and coarsening of Nb and Fe are observed at multi-grain junctions under 700 °C deformation. Further elemental analysis based on Figure 16b,c confirms that the aggregation regions of Nb and Fe elements correspond to β-Zr phases. It can be reasonably concluded that the segregation of β-stabilizing elements (Nb and Fe) effectively elevates the thermodynamic driving force for the α→β phase transformation. Accordingly, β-Zr preferentially nucleates at multi-grain junctions and grain boundaries and subsequently grows inward into adjacent α grain matrices.

## 5. Conclusions

(1) Hot compression experiments were implemented to characterize the plastic flow characteristics of the Zr-Nb alloy under varying thermal and strain-rate conditions. The results reveal that the flow stress of the Zr-Nb alloy exhibits a negative correlation with deformation temperature and a positive correlation with applied strain rate, declining with rising temperature and reduced strain rate. The calculated hot deformation activation energy of the tested Zr-Nb alloy is 281.5228 kJ/mol. On the basis of the acquired rheological curves under diverse hot compression parameters, the constitutive equation of the alloy is established and presented as follows:

$$\sigma =\frac{1}{0.00787091}\ln\{{\left(\frac{Z}{1.69693\times 10^{14}}\right)}^{\frac{1}{5.779984}}+{\left[{\left(\frac{Z}{1.69693\times 10^{14}}\right)}^{\frac{2}{5.779984}}+1\right]}^{\frac{1}{2}}\}$$

(2) Based on the constructed hot processing map, the optimal processing window for the Zr-Nb alloy is determined. The desirable hot deformation parameters cover a temperature range of 650~900 °C and a strain rate range of 0.01~1 s^−1^.

(3) Elevated strain rates induce continuous grain refinement and gradually raise the relevant grain size parameter. Compared with the microstructure deformed at 500 °C, the average grain size increases noticeably at 600 °C. Further temperature elevation from 600 to 700 °C contributes to a stabilized grain size distribution with negligible dimensional variation. For deformation temperatures exceeding 600 °C, the majority of grains exhibit a preferred crystallographic orientation along the <0001>//RD. Microstructural characterization confirms that CDRX acts as the dominant recrystallization mechanism during hot compression of the Zr-Nb alloy, while DDRX can also be detected as a secondary microstructural evolution behavior.

(4) Microstructural observations indicate that minor secondary phases remain detectable at a deformation temperature of 600 °C. Intensive segregation of Nb and Fe occurs extensively at grain boundaries at this temperature, manifesting the preliminary features of thermally induced phase transformation. Meanwhile, radially distributed β-Zr grains are generated at multi-grain junctions, representing a prominent microstructural evolution characteristic under high-temperature deformation conditions.

## Figures and Tables

**Table 1:** Chemical composition of Zr-Nb alloy (wt.%).

Material	Nb	Fe	S	Cu	Zr
Zr-Nb alloy	≤1.1	≤0.22	≤0.003	≤0.02	Balance

## Data Availability

The original contributions presented in this study are included in the article. Further inquiries can be directed to the corresponding author.
